# Attendance of Underserved Populations at Field-Based Health Services Events: Application of Quasi-Experimental Methods that Accommodate the COVID-19 Pandemic

**DOI:** 10.1007/s11121-025-01769-z

**Published:** 2025-01-20

**Authors:** Leslie D. Leve, David S. DeGarmo, Jacob Searcy, Elizabeth L. Budd, Jorge I. Ramírez García, Anne Marie Mauricio, William A. Cresko

**Affiliations:** 1 Prevention Science Institute, University of Oregon, 1600 Millrace Dr., Suite 109, Eugene, OR USA; 2 Department of Counseling Psychology and Human Services, University of Oregon, Eugene, OR USA; 3 Cambridge Public Health, University of Cambridge, Cambridge, UK; 4 Department of Data Science, University of Oregon, Eugene, OR USA; 5 Institute of Ecology and Evolution, University of Oregon, Eugene, OR USA; 6 Knight Campus for Accelerating Scientific Impact, University of Oregon, Eugene, OR USA

**Keywords:** COVID-19 testing, Community participation, Latino, Implementation science

## Abstract

The COVID-19 pandemic disrupted the ability to receive health care services. Field-based health services became a logistically feasible alternative to medical center-based care. We compared two different field-based approaches to the delivery of SARS-CoV-2 testing and health education services for Latine communities using a quasi-experimental design that included propensity score matching to accommodate the challenges posed to research by the pandemic. From September 2021 through October 2022, we held 434 testing events, of which 234 used a geolocation approach and 200 used a partner-located approach to determine the location of the health services (*n* = 68 field sites in Oregon). We hypothesized that partner-located sites would obtain higher numbers of tests collected relative to geolocated sites, and that longer drive times to testing sites would be associated with lower testing rates. There were no differences in the number of tests collected by geolocated versus partner-located sites, controlling for population size and time-varying pandemic vulnerabilities measured as COVID-19 cases and deaths. Prior to propensity score weighting, a longer drive time to the testing site (both site types) was associated with a lower likelihood of total tests (IRR = .87, *p* < .01, CI [0.54, 0.92]), of Latine tests (IRR = .69, *p* < .001, CI [0.56, 0.84]), and of male tests collected (IRR = .67, *p* < .05, CI [0.47, 0.94]). The site’s number of prior tests was associated with a significant 2% increase in tests collected and the prior week’s number of county deaths was associated with a roughly 30% decrease in the likelihood of tests collected. However, the reduced testing rate when the death rate was higher was less likely in geolocated sites (IRR = 1.55, *p* < .001, CI [1.20, 2.01]). Implications for the utility of propensity score matching and time-varying covariates to accommodate pandemic challenges posed to research are discussed. Clinicaltrials.gov registration number: NCT05082935. Date of registration: 10/15/2021.

The COVID-19 pandemic did not impact all communities equally. Throughout the first year and a half of the pandemic, communities with a greater density of minoritized ethnic and racial groups suffered from disproportionally higher positive case counts and death rates than communities that included a greater density of non-Latine White individuals (Chen and Krieger, 2021; Lundberg et al., 2023; Mude et al., 2021). In addition, as the pandemic evolved and the availability of vaccines aimed at preventing the spread of SARS-CoV-2, the virus that causes COVID-19, increased in early- to mid-2021, racial and ethnic disparities in vaccine rates were evident (Kriss et al., 2022), further necessitating the urgency of making testing accessible to individuals in minoritized communities. Nonetheless, the vaccine disparity waned over time and by the end of November 2021, disparities in vaccination coverage for some racial and ethnic groups narrowed, with coverage being similar for Latine (81.3%), Black (78.2%), Native Hawaiian and other Pacific Islander (75.7%), and non-Latine White (78.7%) adults (Kriss et al., 2022). The wave-like nature of the pandemic, timing of vaccine accessibility, differential rates of infection by racial and ethnic groups, and differential prevention and mitigation strategies used by cities proximally located to one another (e.g., Walsh et al., 2021; Zhao et al., 2023) posed fundamental methodological challenges for research with underserved communities. For example, if services (e.g., health and education) in one region were closed yet services in another region were not, and research participants in an intervention study resided across multiple regions, there could be unintended impacts on the data and outcome analyses.

We sought to address these methodological challenges using a quasi-experimental design that included pandemic-related time-varying covariates and used propensity-score matching to accommodate the challenges posed to research by the pandemic. We demonstrate the utility of this methodological approach within a testing and health education intervention delivered from September 2021 to October 2022 that focused specifically on increasing SARS-CoV-2 testing, (hereafter referred to as “COVID-19 testing”) and health education in multiple Latine communities across the state of OR, USA. We use the term Latine throughout this manuscript for parsimony and to be more gender-inclusive of this widely diverse ethnic population (Miranda et al., 2023). Two key variables were expected to affect participation in COVID-19 testing: (1) the approach for selecting health services field sites to provide free testing and health education, for which the two study arms were geolocating sites that occurred on a recurring and predictable basis or mobilized sites that occurred at varied locations and times based on community partner input, and (2) the drive time for participants to reach their testing site. We apply propensity score matching to sites based on population size and social disparities to illustrate how this methodological tool can address unplanned regional differences associated with the pandemic (i.e., that relate to differences in health and educational services access).

## Approaches for Locating Field-Based Health Services During the Pandemic

When the COVID-19 pandemic struck, there was a need and desire for communities to provide testing at field sites located outside of traditional medical settings. However, public health lacked an empirical basis to help determine the best approach to selecting testing venues, including whether testing should be in fixed, regular locations or should be mobilized to provide access in varied locations frequented by the focal population. To our knowledge, there are no empirical evaluations of which approach would be more effective in reaching the intended population to receive preventative health services. This research gap may be attributable to difficulties in quickly moving preventive health services to different field sites, coordinating research staff deployment, and coordinating the location with community teams who are knowledgeable about the focal population and their accessibility preferences.

We conducted a prior study (phase I) between February 1 and August 31, 2021, where we hosted free COVID-19 testing and provided health education in field sites that were selected using a geolocation approach (Searcy et al., 2023). Specifically, we selected testing locations with a census-driven model using geo-mapping that prioritized establishing testing locations with the greatest density of Hispanic individuals residing nearby. In that study, testing events recurred predictably every 2 weeks at the same time and location. Sites were randomized to either receive the *Promotores de Salud* intervention, which included culturally tailored outreach that encouraged community members to visit the field site for testing and then a brief health education intervention on site during the testing events (Budd et al., 2022), or, to be a waitlist control site that received outreach and health education services as usual. The study tested the effectiveness of the *Promotores de Salud* outreach intervention to maximize the number of Latine community members tested. Using a clustered randomized trial involving 394 testing events in nine counties, DeGarmo et al. (2022) found the culturally tailored outreach was effective in testing 3.8 times more Latine individuals (a medium effect size) at sites that were randomized to include *Promotores de Salud* outreach strategies, compared to waitlist control sites that were randomized to operate “as usual.”

We completed the phase I study in August 2021. However, when the first COVID-19 vaccinations were made widely available in late spring of 2021, and shortly thereafter the Centers for Disease Control and Prevention’s (CDC) guidelines around mask wearing and physical distancing changed, public interest in COVID-19 testing sharply decreased. Yet, infection and death rates remained elevated, and the Delta and Omicron variant waves of the pandemic soon emerged. The decline in testing rates following the rise in vaccine availability posed an implementation challenge to testing promotion efforts, and it became unclear if census-based geolocation approaches to testing were still an effective way to reach Latine communities. To empirically address this challenge, we conducted a phase II study to test whether an alternative to geolocated field sites, specifically, mobilization of field sites using weekly guidance from community and public health partners to select testing sites, would yield higher rates of attendance at testing events in comparison to geolocated sites. Implementation researchers have long emphasized the importance of community contexts of interventions (Aarons et al., 2011) and bidirectional knowledge transfer between researchers and community partners (e.g., HEALing Communities Study Consortium, 2020; McCreight et al., 2019; Meador et al., 2023). Accordingly, we posited that it would be beneficial to change testing site locations rapidly and strategically during the COVID-19 pandemic based on up-to-date information provided by community partners. Evaluation of different approaches for selecting field site locations for health services during the dynamic context of the COVID-19 pandemic could have implications for service delivery in future public health emergencies.

## Drive Time Can Affect Health Service Accessibility

Algorithmically, selecting potential health services field site locations is a type of facility location problem (FLP; Weber, 1929), which relates to the selection of optimal site locations for facilities serving a population distributed across a geographic area, based on an objective and various constraints. FLPs have been studied extensively in health care (Ahmadi-Javid et al., 2017), and often play a role in identifying locations for medical facilities during the aftermath of an emergency to select optimal locations. Within the context of the COVID-19 pandemic, FLP algorithms were applied to the COVID-19 context to propose new COVID-19 testing laboratories in Nigeria (Taiwo, 2020) and to propose additional support to specific pharmacies for improved testing access (Risanger et al., 2021).

We leveraged FLP approaches to inform site location for phase I of our study. Specifically, we used a machine learning approach to optimize site selection. The algorithm was based on minimizing driving time from Latine population centers to the COVID-19 field site locations where testing and health education would be provided. Guided by FLP approaches, our evaluation indicated that, between February 2021 and September 2021, a drive time optimization index was significantly associated with increased turnout at COVID-19 field testing events among Latine community members (Searcy et al., 2023). Specifically, greater average drive time (scaled in the regression to 7-min units) was associated with 34% less likelihood of Latine individuals getting tested per event (a medium effect size). Given the differential barriers that Latine individuals may face in seeking access to health services (e.g., Pérez-Escamilla et al., 2010), evaluating whether drive time remained an effective predictor of testing once vaccines were routinely available was a second goal of the current study. However, because the two arms of the study (recurring geolocated sites and mobilized partner-located sites) may yield sites that differ in factors such as population size (an indicator of rurality) and social disparities related to the pandemic, a propensity score approach was applied using site ZIP Codes.

## Hypotheses

We posited two primary hypotheses and one exploratory research question. First, after applying propensity score matching on site-level characteristics (e.g., population size and three social disparity indices), we hypothesized that partner-located sites would yield more COVID-19 tests conducted relative to geolocated sites, controlling for time-varying pandemic vulnerabilities. Second, extending findings from phase I of this study regarding drive time (Searcy et al., 2023), we hypothesized that longer drive times to the field-site locations would be associated with fewer COVID-19 tests conducted, irrespective of the field site type. In exploratory analyses, we examined potential two-way interactions among our Level 1 predictors (prior number of samples collected, COVID-19 related cases, and COVID-19 related death) and our Level 2 predictors (study arm, drive time, Latine populace).

## Method

### Study Design and Site-Level Sample

The site-level study design is a clustered quasi-experimental, nonrandomized, comparative effectiveness trial conducted between September 1, 2021 and October 31, 2022 (14 months). There were two study arms that occurred simultaneously: geolocation of testing sites and partner location of testing sites. To address the potential threat of confounds related to the pandemic, the study arms were propensity score matched prior to testing the study hypotheses (see “Analytic Strategy” below). In total, *n* = 481 field-based testing events were scheduled. Of those, 47 were cancelled due to extreme weather conditions including heatwaves, high winds, fire danger, or unsafe air quality (i.e., Air Quality Index > 99). There was no significant difference in cancellation rate by group condition (12% geolocated and 10% partner located), resulting in *n* = 434 total events in the analytic sample (234 geolocated and 200 partner located) clustered within 68 site locations. The average number of repeated testing events per cluster was 6.38 events (*SD* = 8.12) per field site, ranging from 1 to 36. The study period represented the height of both the Delta and Omicron phases of the pandemic, and vaccines were generally widely available and accessible. Study arm characteristics and significant differences prior to propensity score matching are shown in Table 1. This study includes no individual-level data or participants.

**Table 1:** COVID-19 testing event field site characteristics with means, standard deviations, and mean comparisons (prior to propensity score matching)

	**Geolocated site** (*n* = 234 events)		**Partner-located site** (*n* = 200 events)		**Mean** test	
	*M*	*SD*	*M*	*SD*	*t*	
**COVID-19 test samples**						
Total	8.35	14.25	7.73	18.28		0.39
Latine	4.44	6.77	5.838	15.01		1.26
Female	4.75	8.08	4.25	9.78		0.59
Male	3.52	6.35	3.22	8.53		0.42
**Model covariates**						
Lag samples collected	7.94	14.36	6.11	17.88		1.18
Drive Time Index	829.01	255.06	789.17	258.03		1.61
Latine populace/100	39.69	30.97	80.48	65.23		8.10***
Lag log county cases	6.06	1.05	6.03	1.46		0.28
Lag county deaths	7.24	4.99	6.21	5.98		1.97*
**Matching covariates**						
Child Opportunity Index	38.58	16.41	37.36	19.12		0.21
Social Vulnerability Index	0.87	0.06	0.85	0.16		0.45
Pandemic Vulnerability Index	0.54	0.03	0.53	0.03		1.03
Population size/1000	22.43	16.11	29.60	17.37		1.38

^*^p < .05, **p < .01, ***p < .001

#### Recruitment of Individuals to Field Site Testing Events

All field sites in both study arms offered free testing to anyone age 3 and older. There were no other exclusion criteria. Outreach across all testing events in both study arms was delivered by *Promotores*, who were community health workers hired by partner community-based organizations. Outreach aimed to increase attendance of Latine individuals at testing events through a variety of strategies tailored to the respective local Latine community such as the following: promoting testing via social media, emails, and mass texting; in person promotion at locations frequented by Latine community members (e.g., Mexican grocery stores, Spanish-language church services, schools); and advertising in print media and Latine radio stations (Budd et al., 2022). All social media posts, flyers, and print outreach materials were co-designed with community-based organizations and available in English and Spanish. Key Latine community partners were continuously engaged in the project, with weekly or monthly meetings with the Oregon Health Authority, county public health departments, a Community and Scientific Advisory Board, community-based organization leadership, and the *Promotores* to (a) share up-to-date information and resources about the state’s pandemic mitigation strategies, (b) plan testing event locations vis-à-vis other regional COVID-19 mitigation events, and (c) problem solve and continuously share outreach strategies.

#### Anterior Nares and Saliva Sample Collection and Testing

To collect biosamples for testing, anterior nares swabs or saliva samples were self-collected under the guidance of trained study staff, placed in buffering agent, and transported to the molecular laboratory. Diagnostic testing was conducted at the University of Oregon’s CLIA-certified laboratory employing the FDA emergency use authorized (EUA) Thermo Fisher TaqPath qPCR assay (Food & Drug Administration, 2020) and analyzed using the Applied Biosystems COVID-19 Interpretive Software. Communication of test results to participants was done by the lab and was available in Spanish, English, and Mam, with links provided to post-test COVID-19 resources. The testing was completed voluntarily as part of a person’s private health care services. As such, no identifiable data from the testing activities were shared from the lab to the research team and there were no human subjects research participants (i.e., site-level data only).

### Measures

#### Dependent Variable: Number of COVID-19 Tests Collected

The criterion outcome was the number of completed COVID-19 diagnostic samples collected at each testing event. Data were collected in aggregate form and were extracted from the CLIA-certified lab as total number of samples tested, number of samples collected from Latine individuals, and number of samples collected from female and male individuals. Latine ethnicity and female/male indicators were provided by the lab and derived from the participants’ self-report on their medical record. Cross-classified data were not available due to the de-identified nature of the data that the lab provided to the research team (e.g., the number of individuals who identified as female Latine was unknown to the research team).

#### Site Level Characteristics (Level 2)

##### Study Arms: Geolocated or Partner-Located Site

Sites were either coded as “1” for a geolocated site or “0” for a community partner-located site. To select the geolocated sites, the research team first identified counties that had a large percentage of Latine residents. Next, we contacted county public health entities and our Latine Community and Scientific Advisory Board to determine whether each county needed additional testing for Latine community members. If they did, we used a geo-mapping algorithm that incorporated Latine population density to select potential locations for field-based testing sites (Searcy et al., 2023). As a final step, we provided the Community and Scientific Advisory Board and our community partners with a list of site proposals from the geo-mapping algorithm, and final sites were selected in collaboration with community partners. Geolocated sites offered free testing every 2 weeks at the same time and location. For the partner-located arm, sites were selected on an ongoing basis in consultation with community partners based on their perceptions of the site’s ability to attract members of the Latine community on a particular day for a particular event. Partner-selected event locations varied each week and were guided by factors such as the partners’ knowledge of events where Latine individuals may already be traveling to receive services (e.g., a Mexican Consulate mobile event, a market, or a festival). Study arm (geolocated or partner located) was the independent variable for hypothesis 1; both arms received the outreach and intervention.

##### Average Drive Time Index

Average drive times to the testing site were estimated using the Python package OSMnx (Boeing, 2017). OSMnx utilizes the OpenStreetMap database to retrieve surface roads and build a graph with nodes at intersections connected by edges weighted by road distance divided by the associated speed limit. Optimizing average estimated drive time with a specific number of sites per county is generally referred to as a *p* median FLP, where *p* sites are selected to minimize a cost function. The index started with *K* potential site locations for each county, *M* population centers, and *N* testing sites to place in the FLP solution (see Searcy et al., 2023 for more details). Distance *d* was calculated in parallel with each process estimating the shortest drive time on a street graph between a given node geographically closest to the center of a target block group and the node geographically closest to a potential site location. Average drive time was then calculated from all census blocks within a 45-min drive of a given site. This was the independent variable for hypothesis 2.

##### Latine Populace

The covariate for Latine populace was computed as the U.S. census count of the Hispanic populace divided by 100. Data were matched to the site level by ZIP Code.

#### Testing Event Level Characteristics (Level 1)

To account for the dynamic nature of the pandemic over the course of the study and associated factors that might impact testing rates, three relevant time-varying covariates were included across study arms: the prior number of samples collected (to address potential familiarity with or duration of the site), COVID-19 cases, and COVID-19 related deaths.

##### Lagged Number of Samples Collected

The lagged number of test samples collected from the prior site event was included as a control in the analyses.

##### Lagged Number of COVID-19 Cases

The weekly number of new cases was ascertained from public records and was log transformed to help meet the assumption of homogeneity of variance among predictors. In addition, the number of new cases was lagged by one week and matched to the testing site to meet time-ordered causal assumptions (Github, 2022).

##### Lagged Number of COVID-19 Deaths

The number of deaths was ascertained from public records and was lagged by 1 week (Github, 2022).

#### Analytic Strategy

To address potential confounders of the non-randomized design, we computed propensity score (PS) weights for study arms matched to site ZIP Codes. The PS was the conditional probability of being assigned to the geolocated condition, given by the log odds of assignment shown in Eq. 1. Covariates included census data on population size and three social disparity indices obtained from the RADx-UP Coordination and Data Collection Center (RADx-UP CDCC, n.d). First, the Child Opportunity Index (COI 2.0; Acevedo-Garcia et al., 2020) ranged from 1 to 100 is a nationally normed measure of children’s opportunity comprised of three primary census-tract domains: education, health and environment, and social and economic opportunity indicators. Second, the Ssocial Vulnerability Index (SVI; Flanagan et al., 2018) is a percentile ranking comprised of 15 risk indicators clustered into 4 overarching domains: socioeconomic status, household composition and disability, racial/ethnic minority status and language, and housing type and transportation. Third, the Pandemic Vulnerability Index (PVI; Wolkin et al., 2022) is a national percentile score comprised of 12 indicators clustered into 4 overarching domains: COVID-19 infection rate, population concentration, vaccine availability and intervention measures, and health and environment.

1 $$\mathrm{ln}\left(\frac{PS}{1-PS}\right)= {\beta }_{0}+{\beta }_{1}COI+ {\beta }_{2}SVI+ {\beta }_{3}PVI+ {\beta }_{4}Population Size/1000$$

For primary analyses, generalized linear mixed models were specified to address the repeated field site event data and the count outcomes. A negative binomial Poisson model was specified to address overdispersion, specified as the log of the expected number of tests collected as shown in Eq. 2:

2 $$Log[E(SARS-CoV-2 test sample count)] = \gamma 00 + \gamma 01(geolocated vs. partner site event) + \gamma 02(drive time index) + \gamma 03(Latine populace) + \gamma 10(lagged log of COVID-19 cases) + \gamma 20(lagged COVID-19 deaths) + u0 + u1 + u2 + r$$

where the log test count is regressed on the Level 2 site condition contrast *γ*_01_, the drive time index *γ*_02_, census count of Latine populace* γ*_03_, and the Level 1 time-varying weekly COVID-19 cases and deaths in the prior week *γ*_10_ and *γ*_20_, plus random residual terms for the predicted model (*u*_*0*_), Level 1 covariates (*u*_*1,*_* u*_*2*_), and Level 2 covariates (*r*). Analyses were conducted in the *glmer* package in R, estimated with weighted negative binomial models. In addition to log transformation of COVID-19 cases, continuous covariates were standardized in the final prediction model. Finally, potential two-way interactions among predictors were examined.

## Results

**Fig. 1:**
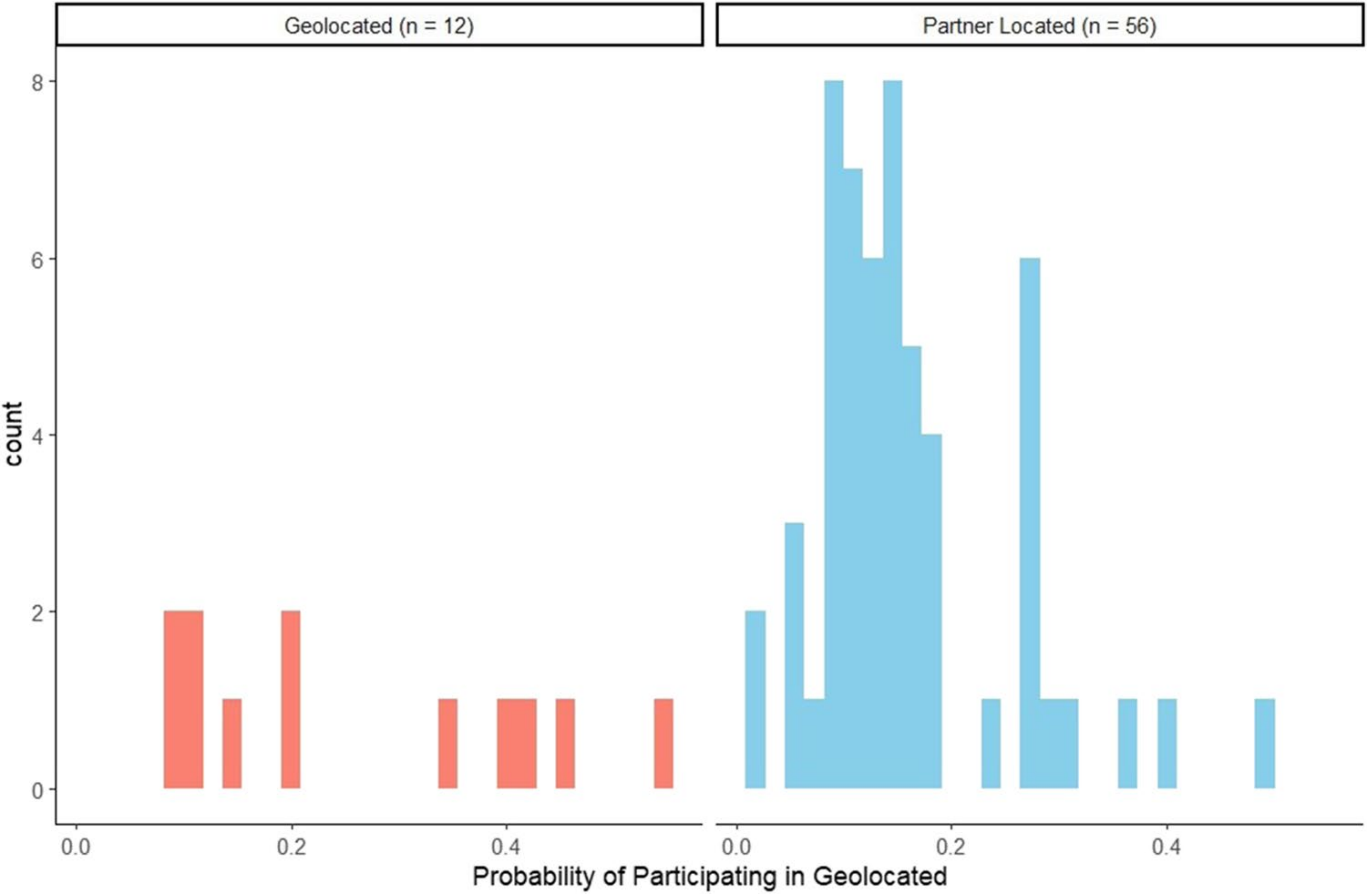
Histogram distribution of propensity score matching of nonrandomized groups. Groups were matched on nationally normed Child Opportunity Index, Social Vulnerability Index, Pandemic Vulnerability Index including vaccinations, and population size

Figure 1 displays the propensity weighting distribution for the geolocated and partner-located study arms. Results of weighted comparative effectiveness models are presented in Table 2 in the form of incident rate ratios (IRR) estimating probabilities of total samples collected and by subgroups for Latine, female, and male. Incident rate ratio (IRR) values > 1 indicate a greater likelihood of a community member receiving a test, and values < 1 indicate a lower likelihood. Only significant effects of the potential two-way interactions are displayed among the continuous predictors and field site location approach.

**Table 2:** Weighted study arm incident rate ratios for multilevel negative binomial model predicting count of COVID-19 tests collected per event (*n* = 434 events and *n* = 68 sites)

	Total test count (*n* = 3531 tests)		Latine test count (*n* = 2228 tests)		Female test count (*n* = 1981 tests)		Male test count (*n* = 1478 tests)		
	IRR	95% CI	IRR	95% CI	IRR	95% CI	IRR		95% CI
Fixed effects									
Intercept	3.31*	[1.32, 8.30]	2.36	[0.97, 5.74]	2.11	[0.92, 4.84]		1.32	[0.51, 3.45]
Lagged tests collected	1.02***	[1.01, 1.02]	1.02***	[1.01, 1.02]	1.02***	[1.01, 1.02]		1.02***	[1.01, 1.02]
Geolocated vs partner	1.82	[0.23, 14.53]	1.37	[0.20, 9.48]	1.64	[0.28, 9.77]		1.90	[0.26, 14.20]
Drive Time Index	0.82	[0.34, 1.91]	0.89	[0.40, 1.98]	0.88	[0.41, 1.89]		0.79	[0.30, 2.07]
Latine populace	1.03	[0.40, 2.67]	0.95	[0.38, 2.38]	1.01	[0.43, 2.39]		1.03	[0.39, 2.77]
Lag log county cases	1.11	[0.83, 1.45]	1.19	[0.87, 1.62]	1.14	[0.81, 1.59]		1.17	[0.79, 1.73]
Lag county deaths	0.68***	[0.56, 0.84]	0.71**	[0.57, 0.88]	0.71**	[0.55, 0.91]		0.69*	[0.52, 0.93]
Two-way interactions									
Geo × county deaths	1.55***	[1.20, 2.01]	1.43*	[1.07, 1.89]	1.47*	[1.07, 2.00]		1.55*	[1.08, 2.22]
Marginal *R*^2^	0.03		0.03		0.04			0.03	

*IRR* incident rate ratio, *CI* confidence interval

^***^p < .001; **p < .01; *p < .05

Hypothesis 1 was not supported by the data. There were no significant differences in testing rates by geolocated versus partner-located sites, controlling for site characteristics and time-varying pandemic vulnerabilities measured as COVID-19 cases and deaths.

Hypothesis 2 was not supported as a main effect for total testing samples collected. However, we note that unweighted models did support a drive time effect; specifically, the greater drive time to a testing site, the lower the likelihood of total sample tests collected (IRR = 0.87, *p* < 0.01, CI [0.54, 0.92]), Latine samples collected (IRR = 0.69, *p* < 0.001, CI [0.56, 0.84]), and male samples collected (IRR = 0.67, *p* < 0.05, CI [0.47, 0.94]). That is, controlling for study arm (geolocated or partner located), drive time was a significant predictor of testing likelihood before propensity score matching was applied. As one might expect, after weighing the nonrandomized sites, this key differentiator of study arms was rendered nonsignificant.

Among covariate main effects, the number of testing samples collected from the previous site event was associated with a significant 2% increase in total testing samples collected, Latine, female, and male (IRR = 1.02, respectively for each category, *p* < 0.001, CI [1.01, 1.02]). Among the time-varying COVID-19 cases and deaths, the number of deaths occurring in the prior week of the testing event was associated with a roughly 30% decrease in the likelihood of sample tests collected (IRR = 0.68, *p* < 0.001, CI [0.56, 0.84]), and similarly for Latine (IRR = 0.71), females (IRR = 0.71), and males (IRR = 0.71).

**Fig. 2:**
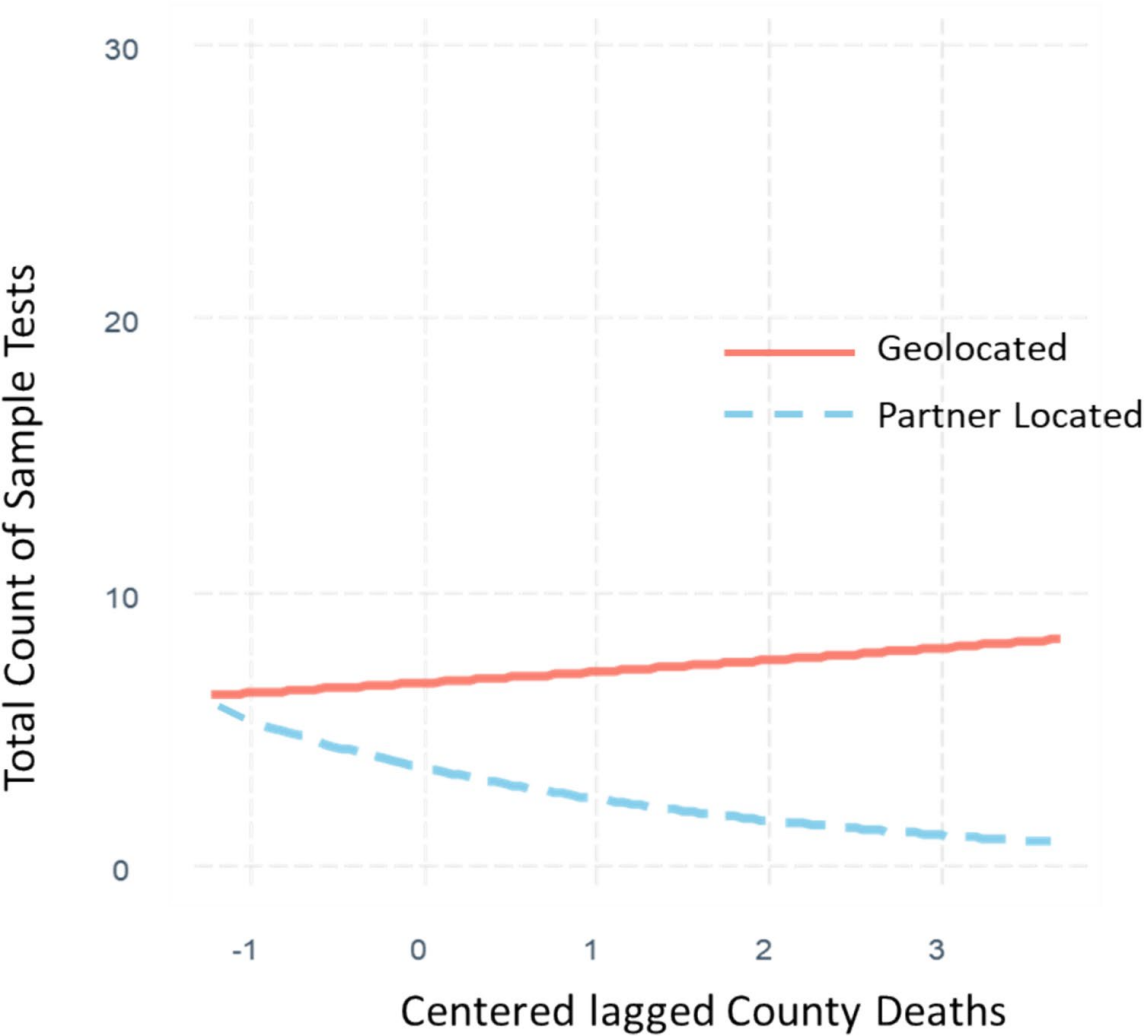
Moderating effect of geolocated testing sites versus partner-located sites on the effect of centered lagged number of county COVID-19 deaths from prior calendar week in predicting total count COVID-19 samples collected per testing event

Among the possible two-way interactions tested, there was a significant moderation of the county deaths effect based on the study arm. The geolocated × county deaths IRR was 1.55 (*p* < 0.001, CI [1.20, 2.01]), representing a moderate effect. That is, geolocated sites were more than one and a half times more likely to collect test samples in the face of higher COVID-19-related deaths than were partner-located sites (see Fig. 2). The effect was consistent for total samples, Latine, males, and females.

## Discussion

The COVID-19 pandemic created a context wherein the need for prevention science research and solutions skyrocketed. Public health was diminishing drastically, as measured by both physical health and mental health indicators (Ernst et al., 2022; Kauhanen et al., 2023; Moreno et al., 2020; Shanbehzadeh et al., 2021), and society needed effective prevention strategies and solutions that could be rapidly deployed in unpredictable and uncertain contexts. Yet, school and health facility closures, the differential impact of the pandemic on minoritized groups, and the differential preventative measures taken within neighboring regions within a county or state posed challenges for intervention research that drew participants from multiple regions. If some participants were more likely to live in a region that was more adversely affected by the pandemic than other participants, and this pandemic-related difference was non-random, the effects of the intervention might be confounded by pandemic factors.

In this report, we empirically examined a research effort that was designed to support the health of individuals from underserved populations during the COVID-19 pandemic by comparing two strategies hypothesized to facilitate access to free testing and health education services in field-based settings: recurring geolocated sites versus mobilized partner-located sites. We incorporated time-varying covariates and applied propensity score matching to account for potential pandemic-related differences in our site locations in the two arms that could confound the understanding of the intervention effects. Our quasi-experimental intervention design indicated that geolocated testing sites remained a successful approach in the middle phase of the pandemic (September 2021–October 2022). Contrary to our prediction, there was no performance advantage for the mobilized partner-located site approach once propensity score matching and time-varying covariates were applied (hypothesis 1); the two arms performed similarly in terms of the number of tests collected. Further, when interactions among predicters were considered, the geolocated sites performed more than 1.5 times *better* than partner-located sites in the face of higher COVID-19 death rates in the region the week prior to the testing event (our exploratory hypothesis). These unexpected findings suggest the importance of retaining health services in locations that are in proximity to the communities where the target population resides and holding them in predictable and routine ways (every 2 weeks at the same day/time/location in this study), especially when death rates are elevated. In the absence of elevated death rates, both arms performed similarly well.

We also identified a main effect of the number of prior tests: sites that performed well in the past continued to perform well thereafter. This finding replicated the main effect of lagged COVID-19 cases from our phase I study that was conducted earlier in the pandemic, before the Delta or Omicron variant waves had onset in Oregon (DeGarmo et al., 2022), and further supports the conclusion that stable and recurring sites may have benefits in terms of the population’s awareness that the site’s health services are a trusted resource for testing.

Analyses examining the benefits of holding testing events near the focal populations’ residences yielded different results when propensity score weights were applied versus not applied. The unweighted models replicated our prior finding that greater drive times were associated with a lower likelihood of testing (Searcy et al., 2023). In the current unweighted analyses, the drive time effect was present for both study arms and occurred during the Delta and Omicron variant phases of the pandemic. This result was consistent with our second hypothesis. However, hypothesis 2 was not supported when propensity score matching was applied. This likely occurred because some key differentiators between the two nonrandomized study arms were included in the propensity score variables, including the population size (e.g., an indicator of rurality) and social disparity indices, and thus the arms were rendered equivalent.

The drive time analyses indicate the potential value of propensity score matching to accommodate differences in the two intervention arms that may be correlated with the effects of pandemic. In our example, propensity score matching dissipated differences thought to be caused by one of our predictors (drive time) on our outcome variable (COVID-19 testing rates). In other studies, it could help control for the effects of nuisance variables caused by differences between participants as a function of disparities in the way the COVID-19 pandemic affected people and that might be correlated with intervention arm or outcomes. For example, in a school-based design, if participants in one school had fewer instructional hours than participants in another school due to pandemic-related school closures, propensity score matching could help disambiguate this COVID-19 nuisance effect from the effects of the intervention.

### Limitations and Future Directions

The study results should be interpreted with some caution. First, this study was not randomized and, therefore, unobserved biases cannot be ruled out in the comparison of effects. Propensity score matching helped to strengthen conclusions, but other unaccounted for differences between the study arms likely impacted results. Related, our design does not allow us to distinguish between the site selection approach (geolocated versus partner located) and the regularity of the field site events, because both varied simultaneously in our study (i.e., partner-located sites changed locations regularly as a function of community partner input, whereas geolocated sites were consistently held every 2 weeks at the same time and location). Thus, a key ingredient may have been the regularity and predictability of the geocoded field site events, rather than their specific geocoded location—this cannot be disambiguated in the current study and is a topic for future research. Further, our study took place in a single state and focused on serving a specific underserved segment of the population (Latine communities), and study results may not be generalizable beyond this sample. Thoughtful community partner engagement is essential to generalize our results to future projects.

In addition, the changing nature of the pandemic required that our team and community partners work together to review and modify our *Promotores de Salud* outreach and health education materials quarterly according to updated national, state, and county-level guidance. The changing nature of the intervention content over the course of a clinical trial was an unfamiliar experience for us as prevention scientists and may have impacted our study outcomes. Because the same modifications were made for all sites in both study arms, we are unable to evaluate the consequences of these modifications on testing rates. We also did not collect sufficient data to conduct a cost-effectiveness analysis, which is an important next step.

The evolving nature of the pandemic required that we include time-varying covariates in our analyses, specifically, the number of prior tests collected, COVID-19 cases, and COVID-19 death rates. The number of prior tests collected and COVID-19 death rates were drivers of testing rates, and although we lagged them in the analyses, longer-term implications of sustained death rates and case counts were not examined in the current study. Nonetheless, our finding highlights the methodological importance of considering both propensity score matching and time-varying covariates when examining the effectiveness of interventions that occurred during the COVID-19 pandemic, to help account for unintended pandemic influences on outcomes.

Finally, given the sharing restrictions of individual health data, we did not have access to identifiable data and could not cross-classify participants by sex and ethnicity nor look at individual-level drive time as a predictor of testing. Access to individual-level data would have allowed greater granularity in our examination of the predictors of testing rates. For example, in addition to the ZIP Code level data on social disparities, we could have included factors such as migrant status, individual drive time or access to public transportation, trust in the testing site, risk aversion, privacy concerns, family-level socioeconomic status, and family health status as predictors of testing, as recommended by our Latine Community and Scientific Advisory Board.

In summary, we found both geolocated and partner-located field sites were effective ways to provide testing to Latine communities during the COVID-19 pandemic, and that a shorter drive time was only a relevant predictor of testing when propensity score matching was not applied to match the sites on population size and social disparity indices. Overall, testing events held at geolocated sites every 2 weeks were more effective than mobilized partner-located sites when COVID-19 death rates in the region were higher. This knowledge can be applied to inform the value of propensity score matching and time-varying covariates when examining intervention outcomes for studies conducted during the COVID-19 pandemic. In addition, the lessons learned in the current study may be relevant to future public health emergencies, as access to health services extends beyond the pandemic and is relevant for a range of preventative health care needs beyond COVID-19 testing.

## Data Availability

Data are shared with the NIH RADx-UP Coordination and Data Collection Center. Our protocols and data are available upon request from the second author, who had full access to all the data in the study and takes responsibility for the integrity of the data and the accuracy of the data analysis.

## Code Availability

Analysis code is available from the second author (DSD) upon request.
